# Implementing Caregiver Training in Health Systems

**DOI:** 10.1093/geroni/igaf122.1188

**Published:** 2025-12-31

**Authors:** Juliana Cuozzo

**Affiliations:** AARP Public Policy Institute, Washington, District of Columbia, United States

## Abstract

The introduction of Caregiver Training Services (CTS) codes in Medicare’s Current Procedural Terminology (CPT) marks a significant step forward in formalizing support and education for family caregivers. In 2024, the Centers for Medicare & Medicaid Services (CMS) allowed Medicare to reimburse practitioners for caregiver training under the Physician Fee Schedule (PFS). Data from Caregiving in the US shows that 50% of caregivers need training, underscoring the significance of this policy change. This body of work explores the implementation and impact of these codes in the first year of their introduction. Our research explores the utilization of five new CPT codes (97550, 97551, 97552, 96202, and 96203) in their first year, as well as the Health Risk Assessment established for caregivers in 2017 (96161), analyzing Medicare claims data to identify trends and gaps in their use. Additionally, qualitative research captures the perspectives of providers and caregivers on the benefits, challenges, and potential improvements of these codes. This work highlights the importance of translating policy into practice to ensure adequate support without creating additional barriers. By examining the practical applications and outcomes of the new CPT codes, we aim to provide insights into optimizing these policy changes for caregivers, patients, and providers.

